# The management and outcomes of placenta accreta, increta, and percreta in the UK: a population-based descriptive study

**DOI:** 10.1111/1471-0528.12405

**Published:** 2013-08-07

**Authors:** KE Fitzpatrick, S Sellers, P Spark, JJ Kurinczuk, P Brocklehurst, M Knight

**Affiliations:** a National Perinatal Epidemiology Unit, University of OxfordOxford, UK; b University Hospitals Bristol NHS TrustBristol, UK; c Institute for Women's Health, University College LondonLondon, UK

**Keywords:** Abnormal placental adherence, haemorrhage, placenta accreta/increta/percreta

## Abstract

**Objective** To describe the management and outcomes of placenta accreta, increta, and percreta in the UK.

**Design** A population-based descriptive study using the UK Obstetric Surveillance System (UKOSS).

**Setting** All 221 UK hospitals with obstetrician-led maternity units.

**Population** All women diagnosed with placenta accreta, increta, and percreta in the UK between May 2010 and April 2011.

**Methods** Prospective case identification through the monthly mailing of UKOSS.

**Main outcome measures** Median estimated blood loss, transfusion requirements.

**Results** A cohort of 134 women were identified with placenta accreta, increta, or percreta: 50% (66/133) were suspected to have this condition antenatally. In women with a final diagnosis of placenta increta or percreta, antenatal diagnosis was associated with reduced levels of haemorrhage (median estimated blood loss 2750 versus 6100 ml, *P *= 0.008) and a reduced need for blood transfusion (59 versus 94%, *P *= 0.014), possibly because antenatally diagnosed women were more likely to have preventative therapies for haemorrhage (74 versus 52%, *P *= 0.007), and were less likely to have an attempt made to remove their placenta (59 versus 93%, *P *< 0.001). Making no attempt to remove any of the placenta, in an attempt to conserve the uterus or prior to hysterectomy, was associated with reduced levels of haemorrhage (median estimated blood loss 1750 versus 3700 ml, *P *= 0.001) and a reduced need for blood transfusion (57 versus 86%, *P *< 0.001).

**Conclusions** Women with placenta accreta, increta, or percreta who have no attempt to remove any of their placenta, with the aim of conserving their uterus, or prior to hysterectomy, have reduced levels of haemorrhage and a reduced need for blood transfusion, supporting the recommendation of this practice.

## Introduction

Three variants of abnormally invasive placentation are recognised: placenta accreta, in which placental villi invade the surface of the myometrium; placenta increta, in which placental villi extend into the myometrium; and placenta percreta, where the villi penetrate through the myometrium to the uterine serosa and may invade adjacent organs, such as the bladder. Placenta accreta, increta, or percreta is associated with major pregnancy complications, including life-threatening maternal haemorrhage, large-volume blood transfusion, and peripartum hysterectomy.^1^–^2^ However, limited data exist to guide the optimal management of this condition. The existing literature consists predominately of case reports, and studies undertaken using retrospective review of medical records, over a number of years in a single or small number of tertiary-care institutions.^3^–^6^ Such studies have a number of limitations, including limited generalisability and a lack of statistical power.

The aims of this study were to prospectively identify a national population-based cohort of women with placenta accreta, increta, or percreta to describe the current management of this condition in the UK, and the associated outcomes for women and their infants, in order to inform future practice guidelines.

## Methods

Cases included all women identified as having placenta accreta, increta or percreta, defined as either placenta accreta, increta, and percreta diagnosed histologically following hysterectomy, or post-mortem, or an abnormally adherent placenta, requiring active management, including conservative approaches where the placenta is left *in situ*. The UK Obstetric Surveillance System (UKOSS) was used to identify cases on a national basis between 1 May 2010 and 30 April 2011.^7^ Every month, report cards were sent to nominated clinicians in each obstetrician-led maternity unit in the UK, with a tick box to indicate the number of cases of placenta accreta, increta, or percreta they had seen that month. The clinicians were asked to return all cards, even when they had ‘nothing to report’. Data collection forms were then sent to the clinicians who reported a case to confirm the diagnosis and request further information concerning potential risk factors, management, and outcomes. All data requested were anonymous, and up to five reminders were sent if data collection forms were not returned. Data were double-entered into a customised database. Information on the women's year of birth and expected date of delivery was used to identify duplicate case reports, and cases were reviewed to ensure that they met the case definition.

A χ^2^ test, Fisher's exact test, or Wilcoxon rank sum test, as appropriate, was used to compare the characteristics, management, and maternal outcomes of the cases according to whether they were suspected of having placenta accreta, increta, or percreta antenatally, and whether an attempt was made to remove any of the placenta around the time of delivery. All analyses were carried out using stata statistical software 11 (StataCorp, College Station, TX, USA).

## Results

During the study period, all 221 UK hospitals with obstetrician-led maternity units contributed data to UKOSS (100% participation) and notified 187 cases of placenta accreta, increta, or percreta, 16 of which were subsequently reported by clinicians as not being cases after all. Data collection forms were received for 144 (84%) of the remaining notified cases: ten were subsequently excluded (four because they were duplicates, three because they delivered outside the study period, and three because they did not meet the case definition), leaving a total of 134 confirmed cases of placenta accreta, increta, or percreta in an estimated 798 634 maternities.^8^,^9^ This represents an estimated incidence of 1.7 per 10 000 maternities (95% CI 1.4–2.0).

### Diagnosis

Placenta accreta, increta, or percreta was suspected prior to delivery in half of the women (66/133, 50%). Twenty-eight (42%) of these women were diagnosed by ultrasound and magnetic resonance imaging (MRI), 32 (48%) by ultrasound only, and six (9%) by MRI only. Table 1 shows the ultrasound and MRI features that were noted. The majority of the women who did not have placenta accreta, increta, or percreta suspected antenatally presented with a difficult or unsuccessful delivery of the placenta, either at vaginal or caesarean delivery (52/65, 80%); other presentations included antepartum haemorrhage (10/65, 15%) and uterine rupture (2/65, 3%).

**Table 1:** Ultrasound/MRI features noted in women who had placenta accreta, increta, or percreta suspected prior to delivery

	Number (%) of cases suspected prior to delivery, diagnosed by ultrasound* (*n* = 60)
Ultrasound features noted**	
Placental lacunae	21 (38)
Loss of clear space	32 (57)
Disruption of bladder–myometrial interface	27 (48)
Increased vascularity	6 (11)
Other	9 (16)

	Number (%) of cases suspected prior to delivery, diagnosed by MRI (*n* = 34)^1^
MRI features noted***	
Uterine bulging	8 (29)
Heterogenous signal intensity within placenta	6 (21)
Dark intraplacental bands	5 (18)
Focal interruptions to myometrial wall	10 (36)
Invasion of pelvic strutures by placental tissue	9 (32)
Other	4 (14)

* Percentage of individuals with complete data.

** A total of 46% (25/56) of cases diagnosed by ultrasound had two or more ultrasound features noted.

*** A total of 36% (10/28) of cases diagnosed by MRI had two more MRI features noted.

Women who had placenta accreta, increta, or percreta suspected antenatally were more likely than those who did not to be multiparous [98% (65/66) versus 84% (56/67), *P *= 0.003], were more likely to have had a previous caesarean delivery [98% (65/66) versus 72% (48/67), *P *< 0.001], and were more likely to have had placenta praevia diagnosed prior to delivery [97% (64/66) versus 33% (22/67), *P *< 0.001]. Sixty-three (95%) of the suspected cases had both placenta praevia diagnosed antenatally and a previous caesarean delivery, compared with 20 (30%) of the unsuspected cases (*P *< 0.001). Of the 20 unsuspected cases who had both placenta praevia and a previous caesarean, one was noted to have no features of morbidly adherent placenta on ultrasound, and one was noted to have an uncertain diagnosis at MRI; the remaining 18 did not appear to have had imaging to specifically look for morbidly adherent placenta.

There was also a suggestion that the women who had placenta accreta, increta, or percreta suspected antenatally had a greater severity of placental invasion, as they were more likely to have a final diagnosis after delivery of placenta increta or percreta, rather than accreta [43% (28/65) of suspected cases versus 27% (18/67) of unsuspected cases, *P *= 0.051]. No other significant differences were found between the antenatally suspected and unsuspected women in terms of the following characteristics: maternal age, ethnicity, socio-economic group, body mass index (BMI), smoking status, gender of infant, or whether the women had a multiple pregnancy, an IVF pregnancy, pregnancy inducted hypertension or pre-eclampsia, other previous uterine surgery, or previous uterine perforation (data not shown).

In total, 65% (87/133) of the women had a final diagnosis after delivery of placenta accreta, 5% (7/133) had a final diagnosis of placenta increta, and 29% (39/133) had a final diagnosis of placenta percreta. The final diagnosis was based on a pathological examination of the uterus for 53% (68/129) of all cases: 48% (41/85) of the placenta accreta cases, 86% (6/7) of the increta cases, and 57% (21/37) of the percreta cases. Among women with a confirmed pathological diagnosis, 60% (41/68) of cases were accreta, 9% (6/68) were increta, and 31% (21/68) were percreta.

### Management and outcomes of placenta accreta, increta, and percreta

**Figure 1:**
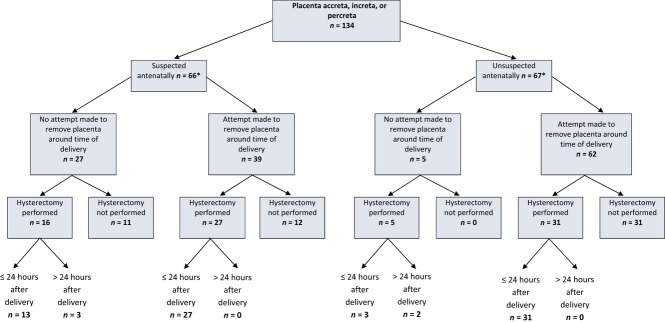
Placenta accreta, increta, or percreta cases, according to whether they were suspected of having this condition antenatally, whether an attempt was made to remove any of the placenta around the time of delivery, and whether a hysterectomy was subsequently performed. *Does not add up to total number of cases—data on whether placenta accreta/increta/percreta suspected antenatally missing for one woman. This woman had an attempt to remove her placenta around the time of delivery and did not have a hysterectomy performed.

Figure 1 shows the cases of placenta accreta, increta, and percreta according to whether they were suspected of having this condition antenatally, whether an attempt was made to remove any of the placenta around the time of delivery, and whether a hysterectomy was subsequently performed. The variety of therapies that were used to prevent and/or treat haemorrhage in the cases is summarised in Table 2. Women who had placenta accreta, increta, or percreta suspected antenatally were more likely than those who did not to deliver by planned caesarean, have no attempt to remove any of their placenta around the time of delivery, have other therapy(ies) to prevent haemorrhage, and be admitted to an intensive therapy unit (ITU)/high-dependency unit (HDU) (Table 3). Although they were less likely to have other therapy(ies) to treat haemorrhage, there was no significant difference in their median estimated total blood loss, the proportion who received a blood transfusion, or the proportion who subsequently had a hysterectomy. Subgroup analysis, however, suggests that although an antenatal diagnosis is not associated with a lower median estimated total blood loss or need for blood transfusion in women with a final diagnosis of placenta accreta [median estimated blood loss 3000 ml (range 300–14 435 ml) in suspected cases versus 3100 ml (range 200–15 000 ml) in unsuspected cases, *P *= 0.9131; 84% (31/37) of suspected cases had a blood transfusion versus 81% (39/48) of unsuspected cases, *P *= 0.761], there is an association in women who had a final diagnosis of placenta increta or percreta [median estimated blood loss 2750 ml (range 250–10 514 ml) in suspected cases versus 6100 ml (range 1500–24 000 ml) in unsuspected cases, *P *= 0.008; 59% (16/27) of suspected cases had blood transfusion versus 94% (17/18) of unsuspected cases, *P *= 0.014].

**Table 2:** Therapies used to prevent and/or treat haemorrhage in women with placenta accreta, increta, or percreta

Therapy	Number (%) of cases that had therapy used to prevent haemorrhage (*n* = 134)	Number (%) of cases that had therapy used for treatment of haemorrhage (*n* = 134)
Syntocinon bolus/IV/IM	12 (9)	4 (3)
Syntocinon infusion	57 (43)	44 (33)
Ergometrine	5 (4)	34 (25)
Prostaglandin F2α	1 (1)	38 (28)
Misoprostol	7 (5)	8 (6)
Intrauterine balloons	5 (4)	28 (21)
B-Lynch or other brace suture	0 (0)	18 (13)
Artery embolisation/balloon tamponade	22 (16)	11 (8)
Pelvic vessel ligation	6 (4)	5 (4)
Intra-abdominal packing	0 (0)	16 (12)
Recombinant activated factor VII	0 (0)	5 (4)
Other	4 (3)	15 (11)

**Table 3:** Peripartum management and maternal outcomes by whether placenta accreta, increta, or percreta was suspected antenatally

Peripartum management/maternal outcome	Number (%), unless otherwise stated, of cases suspected antenatally (*n* = 66)*	Number (%), unless otherwise stated, of cases not suspected antenatally (*n* = 67)*	*P*
**Planned mode of delivery**			
Vaginal	2 (3)	20 (30)	<0.001
Caesarean	64 (97)	46 (70)	
**Attempt made to remove any of placenta around time of delivery**			
No	27 (41)	5 (7)	<0.001
Yes	39 (59)	62 (93)	
**Hysterectomy performed**			
No	23 (35)	31 (46)	0.18
Yes	43 (65)	36 (54)	
**Hysterectomy type**			
Total	25 (58)	18 (50)	0.469
Subtotal	18 (42)	18 (50)	
Other therapy(ies) to prevent haemorrhage**			
No	17 (26)	32 (48)	0.007
Yes	49 (74)	34 (52)	
Other therapy(ies) to treat haemorrhage**			
No	33 (50)	17 (25)	0.003
Yes	33 (50)	50 (75)	
**Median estimated total blood loss in ml (range)**	3000 (250*–*14 435)	3500 (200*–*24 000)	0.126
**Estimated total blood loss (ml)**			
<2500	30 (45)	20 (30)	0.063
2500 or more	36 (55)	47 (70)	
**Blood products given**			
No	17 (27)	10 (15)	0.109
Yes	47 (73)	56 (85)	
Median units of whole or packed red cells transfused (range)***	7 (0*–*24)	7 (2*–*29)	0.783
Median units of fresh frozen plasma transfused (range)***	3.5 (0*–*13)	4 (0*–*12)	0.685
Median units of platelets transfused (range)***	0 (0*–*6)	0 (0*–*4)	0.813
Median units of cryoprecipitate transfused (range)***	0 (0*–*10)	0 (0*–*10)	0.848
Median ml of cell salvaged blood transfused (range)***	75 (0*–*8000)	0 (0*–*1700)	<0.001
**Admission to ITU/HDU**			
No	13 (20)	29 (43)	0.003
Yes	53 (80)	38 (57)	
**Median duration of stay in ITU/HDU in days (range)**	2 (1*–*26)	1.5 (1*–*19)	0.617

* Percentage of individuals with complete data.

** See Table 2.

*** In women who received some type of blood product.

A total of 102 (76%) of the women had an attempt made to remove their placenta around the time of delivery. Sixteen (16%) of these women were noted to have had part (*n* = 12) or all (*n* = 4) of their placenta left in place after the attempt. Fifty-eight hysterectomies were performed following an attempt to remove the placenta, five of which were performed in women who had part and four of which were performed in women who had their entire placenta left after the attempt. Another 21 hysterectomies were performed in women who had no attempt to remove any of their placenta around the time of delivery. Although the hysterectomy rate did not vary according to whether an attempt was made to remove any of the placenta (Table 4), there was a variation in when the hysterectomies were performed: the 58 hysterectomies that followed an attempt to remove the placenta all occurred within 24 hours of delivery (31 known to have been performed within 1 hour of delivery). By contrast, of the 21 hysterectomies that followed no attempt to remove any of the placenta, 16 (76%) were performed within 24 hours of delivery (14 known to have been performed within 1 hour of delivery); the remaining hysterectomies were performed a median of 51 days (range 6–97 days) after delivery because of excessive vaginal bleeding in three cases, uncontrollable vaginal bleeding following a later elective attempt at removal of the placenta in one case, and after discussion with the woman concerned in one case. Of the 11 women who had no attempt to remove any of their placenta and did not subsequently have a hysterectomy, nine (82%) were followed up: the placenta was documented to have completely resorbed in three of these women a median of 145 days (range 134–156 days) after delivery, and six were still awaiting complete resorption at the time of data collection. Of the seven women who did not have a hysterectomy and were noted to have had part of their placenta left after an attempt to remove it, five (71%) were followed up: the placenta was documented to have completely resorbed in four of these women a median of 87.5 days (range 64–144 days) after delivery, and one woman was still awaiting complete resorption at the time of data collection.

**Table 4:** Peripartum management and maternal outcomes of women with placenta accreta, increta, or percreta, by whether an attempt made to remove any of the placenta around time of delivery

Peripartum management/maternal outcome	Number (%), unless otherwise stated, of cases who had no attempt to remove placenta around time of delivery (*n* = 32)*	Number (%), unless otherwise stated, of cases who did have an attempt to remove placenta around time of delivery (*n* = 102)*	*P*
**Caesarean delivery**			
No	2 (6)	14 (14)	0.356
Yes	30 (94)	88 (86)	
**Hysterectomy peformed**			
No	11 (34)	44 (43)	0.379
Yes	21 (66)	58 (57)	
**Hysterectomy type**			
Total	12 (57)	31 (53)	0.771
Subtotal	9 (43)	27 (47)	
Other therapy(ies) to prevent haemorrhage**			
No	7 (23)	42 (42)	0.055
Yes	24 (77)	59 (58)	
Other therapy(ies) to treat haemorrhage**			
No	24 (75)	26 (26)	<0.001
Yes	8 (25)	75 (74)	
**Median estimated total blood loss in ml (range)**	1750 (200*–*15 000)	3700 (500*–*24 000)	0.001
**Estimated total blood loss (ml)**			
<2500	18 (56)	32 (31)	0.011
2500 or more	14 (44)	70 (69)	
**Blood products given**			
No	13 (43)	14 (14)	<0.001
Yes	17 (57)	87 (86)	
Median units of whole or packed red cells transfused (range)***	7 (3*–*24)	7 (0*–*29)	0.597
Median units of fresh frozen plasma transfused (range)***	4 (0*–*13)	4 (0*–*12)	0.763
Median units of platelets transfused (range)***	0 (0*–*4)	0 (0*–*6)	0.583
Median units of cryoprecipitate transfused (range)***	0 (0*–*4)	0 (0*–*10)	0.402
Median ml of cell salvaged blood transfused (range)***	0 (0*–*8000)	0 (0*–*5500)	0.067
**Admission to ITU/HDU**			
No	10 (31)	32 (31)	0.99
Yes	22 (69)	70 (69)	
**Median duration of stay in ITU/HDU in days (range)**	1.5 (1*–*26)	2 (1*–*19)	0.894

* Percentage of individuals with complete data.

** See Table 2.

*** In women who received some type of blood product.

Five of the women who had no attempt to remove any of their placenta were treated with methotrexate: three of these women were amongst those who subsequently had a delayed hysterectomy, one was amongst those whose placenta was documented to have completely resorbed, and one was amongst those still awaiting complete resorption. Of the women who were noted to have had part of their placenta left in place after an attempt to remove it, two were treated with methotrexate: both of these women were amongst those whose placenta was documented to have completely resorbed.

As well as being more likely to have been diagnosed antenatally, in terms of characteristics, women who had no attempt to remove any of their placenta were more likely than those who did to have had a previous caesarean delivery [97% (31/32) versus 80% (82/102), *P *= 0.025], were more likely to have had placenta praevia diagnosed prior to delivery [88% (28/32) versus 57% (58/101), *P *= 0.002], and were more likely to have a final diagnosis of placenta increta or percerta, rather than accreta [71% (22/31) versus 24% (24/102), *P *< 0.001]; no other significant differences were found in other current pregnancy, previous obstetric, or sociodemographic characteristics (data not shown). Despite being more likely to have a greater severity of placental invasion, the women who had no attempt to remove any of their placenta were less likely to have other therapy(ies) to treat haemorrhage, had a lower estimated total blood loss, and were less likely to have a blood transfusion (Table 4).

Although none of the women with placenta accreta, increta, or percreta died, additional severe morbidity was noted in 18 (13%) of the women: ten had damage to their bowel, urinary tract, or bladder (six were women who had an attempt to remove their placenta and had a hysterectomy within 24 hours of delivery, and four were women who had no attempt to remove any of their placenta, three of whom had a hysterectomy within 24 hours of delivery and one of whom had a delayed hysterectomy); three had sepsis (all three had an attempt to remove their placenta, two of whom had a hysterectomy within 24 hours of delivery); three had a vesicovaginal fistula (two had an attempt to remove their placenta and had a hysterectomy within 24 hours of delivery, and one had no attempt to remove any of their placenta and had a hysterectomy within 24 hours of delivery); one had a uterocutaneous fistula that eventually resolved spontaneously (this woman had no attempt to remove any of her placenta and did not have a hysterectomy); three had a thrombotic event; and two had a cardiac arrest.

Four of the women lost or had their pregnancy terminated before 24 weeks of gestation. The remaining 130 women gave birth to a total of 134 infants (126 singletons and eight twins). Just over half (66/130, 51%) of these women delivered prior to 37 weeks of gestation, the majority (62/66, 94%) by caesarean: 70% (43/61) of the caesarean deliveries performed preterm were carried out as an elective procedure (grade 3 and 4 urgency^11^) at a median of 35 weeks of gestation (range 27–36 weeks of gestation), and 30% (18/61) as an emergency (grade-1 or -2 urgency^11^) at a median of 31.5 weeks of gestation (range 24–36 weeks of gestation). The indication for the majority (14/18, 78%) of these emergency caesarean deliveries was antepartum haemorrhage (10/14, 71% had placenta praevia diagnosed antepartum). There were no stillbirths and two early neonatal deaths amongst the 134 infants, equating to a perinatal mortality rate of 14.9 per 1000 (95% CI 1.8–52.8). Although this was double the national rate of 7.5 per 1000,^12^ the difference was not statistically significant (RR 2.0, 95% CI 0.5–7.8), noting the limited statistical power of this comparison. A total of 59 (44%) of the infants were admitted to a neonatal unit.

## Discussion

### Main findings

This prospective population-based study has two main findings. Firstly, in women with a final diagnosis of placenta increta or percreta, antenatal diagnosis is associated with reduced levels of haemorrhage and a reduced need for blood transfusion. Secondly, making no attempt to remove any of the placenta, either in an attempt to conserve the uterus or prior to hysterectomy, is associated with reduced levels of haemorrhage and a reduced need for blood transfusion.

### Strengths and weaknesses

A major strength of our study is its prospective population-based design, not relying on routinely coded data to ascertain cases. In order to fully capture all cases of placenta accreta, increta, and percreta, including cases managed conservatively, we used a case definition that included clinically as well as pathologically defined cases. We cannot therefore be certain that all cases would have been pathologically confirmed; however, we restricted the inclusion of clinically defined cases to those requiring active management. It is thus unlikely that significant numbers of false-positive cases have been included. Another potential limitation is that we cannot be certain that we have ascertained all cases, despite the presence of several reporting clinicians in each hospital, and the active monthly nature of UKOSS case reporting. However, previous studies using UKOSS have suggested high rates of ascertainment.^13^–^14^ Importantly, we have no evidence of a systematic bias in case ascertainment that may affect the validity of our results.

### Interpretation

Antenatal diagnosis of placenta accreta, increta, or percreta allows for early delivery planning, including the availability of a multi-professional team, discussion of the surgical approach to delivery, preparation for invasive management, including hysterectomy if necessary, as well as ensuring sufficient blood products and other supporting therapies are readily available.^15^ In women with a final diagnosis of placenta increta or percreta, we found that an antenatal diagnosis is associated with reduced levels of haemorrhage and a reduced need for blood transfusion. This association may be the result of observed differences in the management of antenatally diagnosed and undiagnosed women: women diagnosed antenatally in our study were more likely than those without antenatal suspicion to have preventative therapies for haemorrhage, and were less likely to have an attempt to remove their placenta. Regardless, our study also demonstrates that more than half of women with placenta accreta, increta, or percreta have a hysterectomy; early diagnosis will allow for the appropriate planning of anaesthetic and surgical resources in the event this is required, and adequate counselling of the women involved. The study shows that currently placenta accreta, increta, and percreta is not diagnosed antenatally in half of cases, and that 30% of undiagnosed cases have a prior caesarean delivery as well as placenta praevia, a group with a high incidence of the condition (around one in every 20 women).^16^ Ultrasound features such as placental lacunae,^15^ and MRI features such as uterine bulging,^17^ have been documented as being suggestive of placenta accreta, increta, and percreta. Our study only collected information on the antenatally suspected cases that were confirmed pathologically or clinically, so we cannot evaluate the reliability of such features for diagnosing placenta accreta, increta, or percreta; however, previous studies suggest that currently there is no completely sensitive and specific antenatal diagnostic technique for the condition.^15^ In view of this, there is an argument for managing the delivery of very high risk women, such as those with a prior caesarean delivery and placenta praevia, as if they have a morbidly adherent placenta.

Debate remains over the optimal management of placenta accreta, increta, and percreta: if the placenta fails to separate after delivery, leaving it in place and proceeding with either a hysterectomy or conservative management, rather than trying to separate it, is currently recommended by the Royal College of Obstetricians and Gynaecologists (RCOG)^18^; the American College of Obstetricians and Gynecologists^19^ currently make no specific recommendations regarding attempted placental separation. Our study supports the RCOG recommendation, with the finding that making no attempt to remove any of the placenta around the time of delivery, in an attempt to conserve the uterus or prior to hysterectomy, is associated with reduced levels of haemorrhage and a reduced need for blood transfusion. We did not observe any significant differences between the characteristics of women who did and did not have an attempt to remove their placenta that could have offered an alternative explanation for this association. Our study suggests that currently only around a quarter of women with placenta accreta, increta, or percreta have no attempt to remove their placenta. Given the limitation of antenatal diagnosis with the possibility of false positives, however, there may be a case for gently trying to remove the placenta before proceeding with a hysterectomy, when there are no obvious signs of placental invasion.

Conservative management of placenta accreta, increta, and percreta, involving leaving the placenta in place around the time of delivery, with the aim of preserving the uterus, is particularly contentious. One of the largest studies (*n* = 167) to have examined maternal outcome after conservative treatment of placenta accreta, increta, and percreta suggested that conservative management can preserve the uterus in 78.4% of women, with a severe maternal morbidity rate of 6%.^20^ In a recent follow-up study, the same authors concluded that a women's subsequent fertility or obstetric outcome does not appear to be compromised by uterine preservation following placenta accreta, increta, or percreta.^21^ However, the authors suggest that women should be advised of the high risk of recurrence in subsequent pregnancies.

Only 16 women in our study appear to have had no attempt to remove their placenta, in a clear attempt to preserve their uterus. Although preservation of the uterus succeeded in 11 (73%) of these women, five underwent hysterectomy a median of 51 days (range 6–97 days) after delivery: in four of these women this was because of excessive vaginal bleeding. This highlights one of the concerns about conservative management: that women may continue to be at risk of severe bleeding for several months after delivery. Another concern about conservative management is that it may increase a woman's risk of infection. Sepsis was only noted in three women in our study, none of whom were managed conservatively; however, the small number of women managed conservatively makes it impossible to infer that there is a genuinely low risk of sepsis, and further research is needed to address this. Similarly, very few women were managed with methotrexate: we thus have no clear evidence of any added benefit of using this approach.

## Conclusion

In women with a final diagnosis of placenta increta or percreta, an antenatal diagnosis is associated with reduced levels of haemorrhage and a reduced need for blood transfusion, possibly because antenatally diagnosed women are more likely to have preventative therapies for haemorrhage, and are less likely to have an attempt made to remove their placenta. Additionally, more than half of women with placenta accreta, increta, or percreta have a hysterectomy; early diagnosis will allow for the appropriate planning of anaesthetic and surgical resources in the event this is required, as well as adequate counselling of the women involved. However, many cases of placenta accreta, increta, and percreta are currently not diagnosed antenatally, despite the presence of risk factors. Further research is needed to establish the most sensitive and specific antenatal diagnostic techniques. Women with placenta accreta, increta, or percreta who have no attempt to remove any of their placenta, with the aim of conserving their uterus, or prior to hysterectomy, have reduced levels of haemorrhage and a reduced need for blood transfusion, supporting policies that recommend this practice.

### Disclosure of interests

The authors declare that no competing interests exist.

### Contribution to authorship

KF coded the data, carried out the analysis, and wrote the first draft of the article. SS contributed to the design of the study and writing of the article. PS assisted with data coding, conducted validation of the data, and contributed to the writing of the article. JJK contributed to the design of the study and the writing of the article. PB contributed to the design of the study and writing of the article. MK designed the study and supervised the data collection and analysis, and contributed to writing the article.

### Details of ethics approval

This study was approved by the London Research Ethics Committee (ref. 10/H0717/20).

### Funding

This article presents independent research funded by the National Institute for Health Research (NIHR) under the ‘Beyond maternal death: Improving the quality of maternity care through national studies of “near-miss” maternal morbidity’ programme (programme grant RP-PG-0608-10038). The views expressed in this publication are those of the author(s), and not necessarily those of the NHS, the NIHR, or the Department of Health. The funder had no role in the study design, data collection and analysis, decision to publish, or preparation of the article.

## Commentary on ‘The management and outcomes of placenta accreta, increta, and percreta in the UK: a population-based descriptive study’

Placenta accreta spectrum (including accreta, increta, and percreta) is one of the scariest conditions that obstetricians will face. As a relatively infrequent condition, most data regarding placenta accreta are derived from small retrospective case series obtained in single institutions. Thus, the optimal management remains uncertain. In this issue of *BJOG*, Fitzpatrick and colleagues report on 134 women with placenta accreta spectrum in a prospective population-based study in the UK, confirming the dramatic morbidity associated with the condition. Importantly, they noted that antenatal diagnosis was associated with reduced levels of haemorrhage and a reduced need for blood transfusion. Another key observation was that women who had a hysterectomy with no attempt to remove the placenta experienced reduced haemorrhage and a lower rate of blood transfusion than those for whom placental removal was attempted. This is especially impressive, as women with no attempt at placental removal had more severe placental invasion than those with attempts at placental removal.

These findings underscore the importance of antenatal diagnosis. In the current study, only 50% of cases were diagnosed antenatally. Strikingly, 20 of the unsuspected cases had BOTH a previous caesarean delivery and a previous placenta praevia. Such risk factors should prompt clinicians to refer women for evaluation and counselling for possible placenta accreta. In addition to avoiding placental removal, antenatal diagnosis allows for the use of other strategies intended to minimise haemorrhage. Indeed, maternal outcomes are improved when delivery occurs in a tertiary care centre with a multidisciplinary team experienced in the management of placenta accreta (Eller et al., *Obstet Gynecol* 2011;117:331–337).

The rate of placenta accreta in this population-based study was 1.7 per 10 000 maternities, which is considerably lower the rates noted in recent US studies. This may be because of the historically lower rate of caesarean delivery in the UK compared with the US. As the rate of caesarean delivery in the UK has been increasing, it will be of interest to see whether the rate of accreta increases as well. Hopefully the UK Obstetric Surveillance System (UKOSS) used in the current study will provide such data. Also, it is crucial to investigate the possibility that differences in caesarean techniques in the UK and US contribute to different rates of accreta.

We congratulate Drs Fitzpatrick and colleagues for their important contribution to our understanding of the accreta spectrum. Clinicians should be alert to risk factors for accreta and refer such women for antenatal evaluation and counseling. Ideally, women with suspected accreta should undergo scheduled delivery in a centre with expertise and experience in the management of accreta. In most cases, patients should have a planned caesarean hysterectomy, with no attempts at placental removal. Having a well-stocked blood bank is a must. In addition, there are numerous unanswered questions regarding the optimal management of accreta. It is only through more prospective multicentre efforts such as that completed by the UKOSS that we will successfully decrease the morbidity of placenta accreta. Finally, these data illustrate the need to redouble our work to safely reduce caesarean delivery rates.
